# Orbital subperiosteal hematoma associated with frontal and ethmoidal sinusitis

**DOI:** 10.1186/s12886-022-02324-y

**Published:** 2022-03-03

**Authors:** Jonghyun Kim, Hyunkyu Lee, Sehyun Baek

**Affiliations:** Department of Ophthalmology, Guro Hospital, Korea University College of Medicine, 148 Gurodong-ro, Guro-gu, Seoul, 08308 Korea

**Keywords:** Ethmoidal sinusitis, Frontal sinusitis, Orbital subperiosteal hematoma, Superior orbitotomy

## Abstract

**Background:**

We report a rare case of orbital subperiosteal hematoma associated with frontal and ethmoidal sinusitis. Common concerns involving the orbital subperiosteal space include abscess, hematoma and tumor.

**Case presentation:**

A patient presented to our clinic with periorbital swelling and limited extraocular muscle movement in her left eye. Computed tomography revealed a superior subperiosteal mass with frontal and ethmoidal sinusitis. We diagnosed the patient with subperiosteal hematoma and surgical evacuation was performed via superior orbitotomy. Brown serous discharge was drained and biopsy demonstrated fibrin clots. The final diagnosis was orbital subperiosteal hematoma and the patient was discharged with symptoms resolved.

**Conclusion:**

Orbital subperiosteal hematoma is difficult to distinguish from abscess owing to its rarity and similar presentation. Computed tomography is helpful in diagnosis, and surgical evacuation during the early stages is essential to achieving a good outcome.

## Background

The orbital subperiosteal space is a space between the periorbita and orbit that is susceptible to a variety of pathologies including abscess, hematoma and tumor [1]. Although surgery is typically required due to associated pain and diplopia or the need for biopsy, accurate preoperative and postoperative diagnosis is crucial for reducing complications. In particular, orbital subperiosteal hematoma should be distinguished from abscess based on symptoms and radiologic findings. Orbital subperiosteal hematoma can be divided into traumatic and non-traumatic types [2]. The most common cause of orbital subperiosteal hematoma is direct facial trauma [3]. Non-traumatic orbital subperiosteal hematoma is associated with sudden elevation of cranial venous pressure and venous congestion (coughing, vomiting, scuba diving and Valsalva maneuver), systemic diseases with bleeding coagulopathy (scurvy and liver diseases) and paranasal sinusitis [2, 3].

Compared with other causes, orbital subperiosteal hematoma is very rarely reported in association with sinusitis. Patients with trauma and venous congestion generally have a relevant history and bleeding tendency can be determined from blood tests. However, clinicians should consider sinusitis when presented with patients with unknown causes of orbital subperiosteal hematoma.

We report one rare case of orbital subperiosteal hematoma associated with frontal and ethmoidal sinusitis and describe the characteristics, diagnostic tools and treatment of this disease.

## Case presentation

**Fig. 1:**
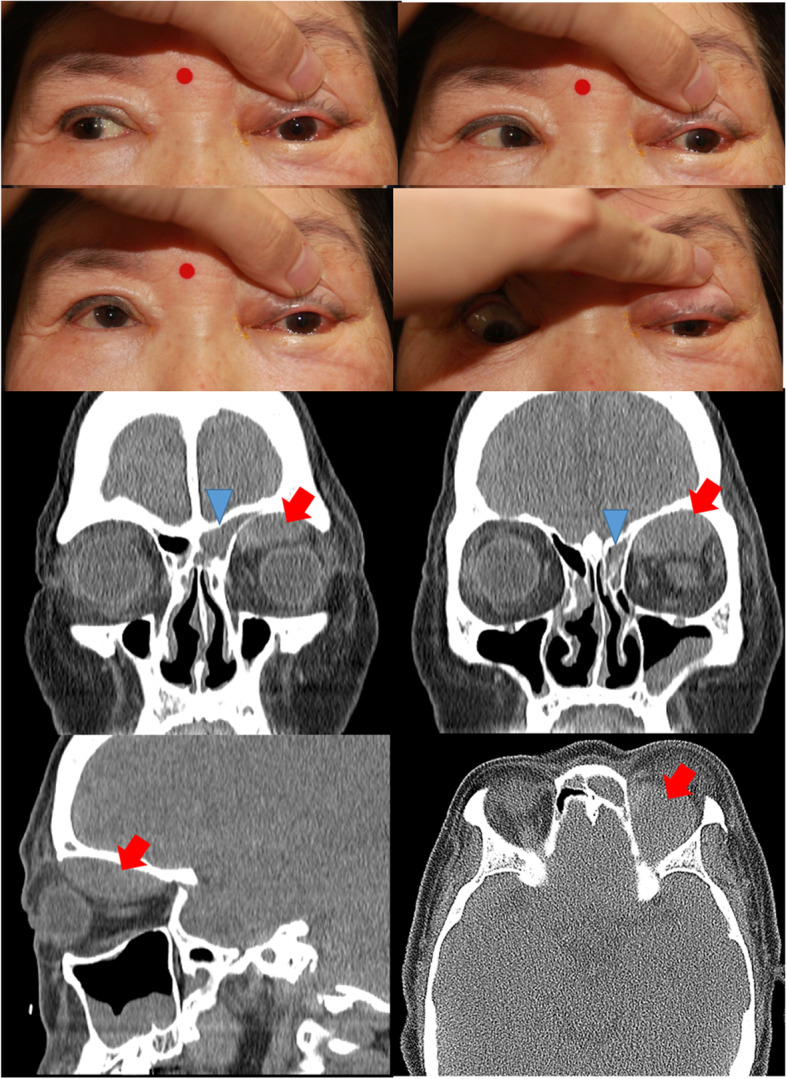
Limited extraocular muscle movement in terms of elevation, depression and adduction of the left eye. A well-demarcated biconvex non-enhancing soft tissue density lesion can be seen in the left superior subperiosteal area on non-enhanced CT

A 70-year-old woman visited our clinic due to periorbital swelling with mild ocular pain of the left eye for 4 days (Fig. 1). She had no history of trauma and ophthalmological surgery. She had hypertension but no bleeding disorders. She had no fever and upper respiratory infection symptoms. Visual acuity was corrected to 20/20 in her right eye and 10/20 in her left eye. Intraocular pressure was 27 mmHg in the left. Elevation, depression and adduction were severely limited in the left eye (Fig. 1). Hertel exophthalmometry demonstrated 4.5mm of proptosis in the left eye. The pupils were isocoric and there were no relative afferent pupillary defects in either eye. Erythrocyte sedimentation rate(ESR) was slightly elevated at 44 mm/h. Coagulation profile and blood glucose level were normal.

Computed tomography(CT) revealed a well-demarcated biconvex non-enhancing soft tissue density lesion in the left superior subperiosteal area (Fig. 1). There was opacification of the frontal and ethmoidal sinus containing hyperdense material with bony thinning. Superior orbitotomy was performed via an eyebrow incision. Brown serous discharge was completely drained from the left superior subperiosteal space.

**Fig. 2:**
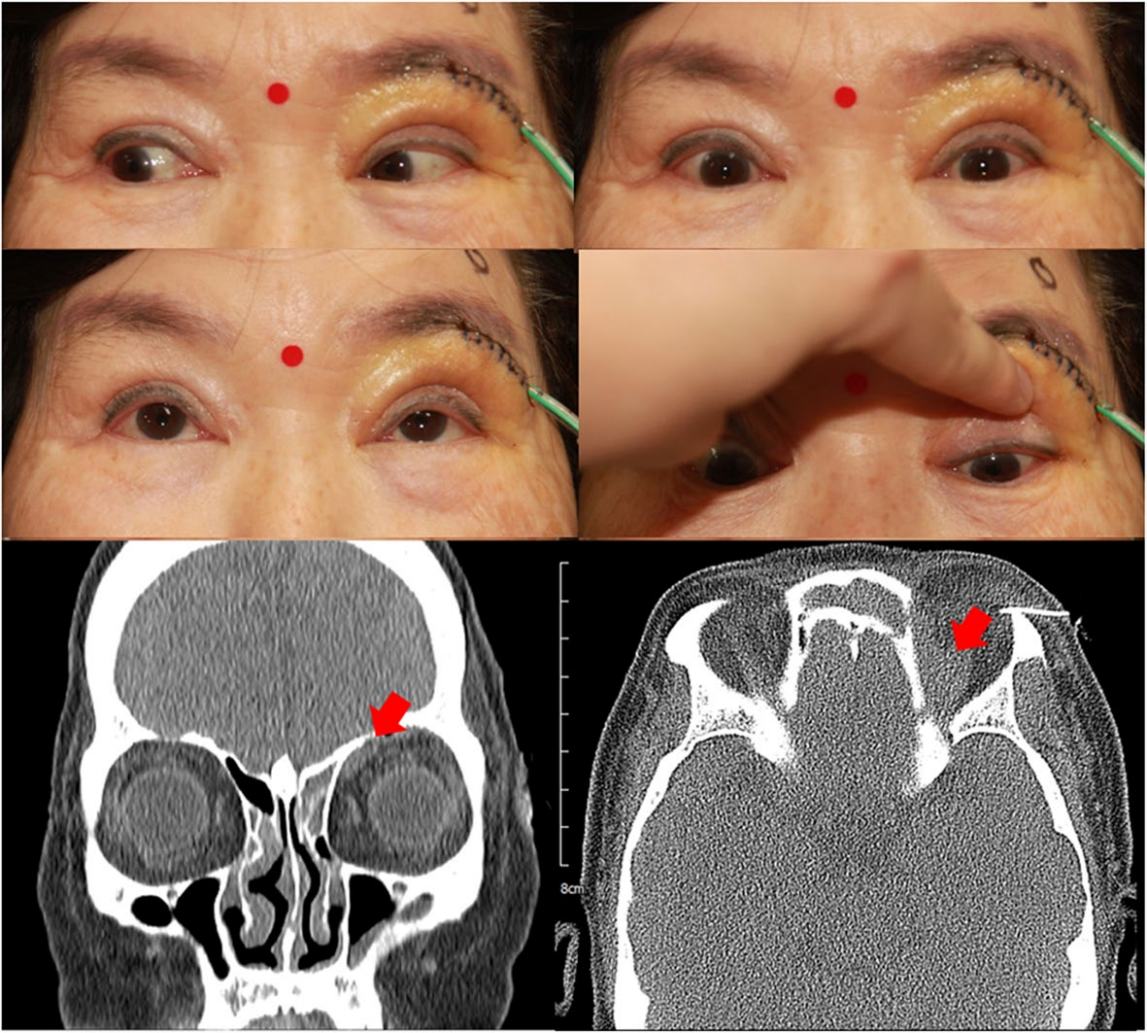
Photograph of the patient, postoperative day 3. Extraocular muscle movement in elevation, depression and adduction of the left eye is normal. Orbital subperiosteal hematoma has decreased in size on non-enhanced CT, postoperative day 4

On postoperative day 3, there were no limitations on left eye movement (Fig. 2). Visual acuity was elevated to 16/20 in her left eye. CT revealed a decrease in the size of the subperiosteal lesion (Fig. 2). The brown serous discharge was made up of fibrin clots without any microorganismal growth. Final diagnosis of orbital subperiosteal hematoma associated with frontal and ethmoidal sinusitis was reached. We considered that thrombophlebitis with venous stasis induced hematoma in subperiosteal space. For chronic sinusitis treatment, we considered oral antibiotics, intranasal steroids and pseudoephedrine and nasal irrigation with normal saline. We referred this patient to otorhinolaryngology for further evaluation and treatment.

This study was approved by the institutional review board of Korea University in Seoul, Korea. The research and data collection were conducted in accordance with the tenets of the Declaration of Helsinki from the Word Medical Association.

## Discussion and Conclusion

Orbital subperiosteal lesions are most commonly abscesses associated with sinusitis, but should be differentiated from other causes such as subperiosteal hematoma, mucocele or malignancy. In one study of subperiosteal masqueraders and abscesses, the abscess group was younger than the masquerader group [1]. The most simple and important differentiation point is a history of recent upper respiratory infection, symptoms of fever and signs of leukocytosis or elevation of inflammation markers like ESR and CRP. Although these clinical signs are suggestive, they are not definitive for either abscess or masqueraders, as seen in our case. Thus, beyond this clinical presentation, radiographic features, particularly radiodensity, may help distinguish subperiosteal abscess from other lesions. Abscesses have radiodensities ranging from 30 to 46 Hounsfield units (HUs) while masqueraders range from 40 to 135 HUs [1].

Sinus and orbital CT is the imaging modality of choice. When comparing hematoma with abscess, coagulated blood is typically seen as hyperdense, while abscesses have a relatively low density. Smooth, well-defined lesions with no periorbital fat stranding suggest a diagnosis of subperiosteal hematoma rather than abscess [4]. Magnetic resonance imaging (MRI) is useful when CT findings are indeterminate, and can increase diagnostic accuracy. MRI is more diagnostic than CT, especially for identifying different stages of bleeding. However, imaging findings are dependent on the effects of blood degradation, and signal intensity on MRI changes over time [4].

In our case, the patient had no history of trauma or recent upper respiratory infection, no fever, no leukocytosis and slightly elevated ESR. CT suggested that frontal and ethmoidal sinusitis with bone thinning. Intraoperative findings and biopsy revealed the lesion to be a hematoma. Fortunately, hematoma was not re-occurred for 3 years of follow up. She was referred to local ophthalmological clinic due to long distance of our hospital from her house.

Most orbital subperiosteal hematomas are post-traumatic, although some can be associated with non-traumatic causes. In traumatic cases, damage to the small vessels under the periosteum creates the hematoma [5].However, in cases of associated sinusitis, sinus infections can extend to adjacent venous systems [2].Compared to arteries, lymphatics and bony structures, veins are the major pathway in the spread of infections [2]. Inflammatory reactions of the sinus can cause venous stasis and thrombophlebitis [4]. Phlebitis in the sinus mucosa can extend to veins in the perioribita, resulting in rupture of the vessels [2, 5]. Orbital hematomas may then undergo suppurative change leading to orbital abscess [5].

In most reported subperiosteal hematoma cases, sudden onset of proptosis and diplopia and ocular pain are common [2]. With severe sinusitis, patients can present with an upper respiratory infection. However, asymptomatic sinusitis can also cause orbital subperiosteal hematoma, as seen in our case. Thus, we should suspect sinusitis even in patients without upper respiratory infection symptoms who exhibit a non-traumatic orbital subperiosteal hematoma.

The treatment of orbital subperiosteal hematoma depends on the severity of orbital compression. There are three treatment options: observation for spontaneous resolution, needle aspiration and surgical evacuation [2]. Some recommend observation in patients with minimal visual loss or mass effect [5]. However, observation is not a suitable treatment for patients with a risk of optic nerve compression; prompt drainage is important to avoid optic nerve involvement and severe vision loss. Needle aspiration has been suggested as a safe and simple treatment method. We assumed ours was a case of sinusitis-associated hematoma and that the lesion could have been present for longer than a few weeks. After consolidation of a hematoma, needle aspiration is ineffective for removal. Surgical evacuation with an incision around the eyebrow and superior orbitotomy is generally recommended, especially in cases associated with sinusitis [5]. Surgical evacuation confirms the diagnosis, enables drainage and prevents recurrence with complete removal.

In conclusion, orbital subperiosteal hematomas are difficult to distinguish from abscesses owing to their rarity and similar presentations. We describe a rare case of orbital subperiosteal hematoma associated with frontal and ethmoidal sinusitis.

## Data Availability

Data sharing is not applicable to this article as no datasets were generated or analyzed during the current study

## Abbreviations

CT: Computed tomography
MRI: Magnetic resonance imaging
HU: Hounsfield units
ESR: Erythrocyte sedimentation rate
CRP: C-reactive protein
CML: Chronic myeloid leukemia
